# Consensus statement of the Italian society of colorectal surgery (SICCR): management and treatment of hemorrhoidal disease

**DOI:** 10.1007/s10151-020-02149-1

**Published:** 2020-01-28

**Authors:** G. Gallo, J. Martellucci, A. Sturiale, G. Clerico, G. Milito, F. Marino, G. Cocorullo, P. Giordano, M. Mistrangelo, M. Trompetto

**Affiliations:** 1 Department of Surgical and Medical Sciences, University “Magna Graecia” of Catanzaro, Catanzaro, Italy; 2 Department of Colorectal Surgery, Santa Rita Clinic, Vercelli, Italy; 3 Department of General, Emergency and Minimally Invasive Surgery, Careggi University Hospital, Florence, Italy; 4 Proctological and Perineal Surgical Unit, Cisanello University Hospital, Pisa, Italy; 5 Department of General Surgery, Tor Vergata University, Rome, Italy; 6 Operative Unit of General Surgery, IRCCS de Bellis, Castellana Grotte, Bari, Italy; 7 Department of Surgical, Oncological and Stomatological Disciplines, University of Palermo, Palermo, Italy; 8 Department of Colorectal Surgery, Whipps Cross University Hospital, Barts Health, London, UK; 9 Department of General and Minimally Invasive Surgery, University of Turin, Turin, Italy

**Keywords:** Hemorrhoids, Hemorrhoidal disease, Conservative treatment, Office-based procedures, Surgical treatment, Special conditions, Postoperative complications

## Abstract

Hemorrhoidal disease (HD) is the most common proctological disease in the Western countries. However, its real prevalence is underestimated due to the frequent self-medication.

The aim of this consensus statement is to provide evidence-based data to allow an individualized and appropriate management and treatment of HD. The strategy used to search for evidence was based on application of electronic sources such as MEDLINE, PubMed, Cochrane Review Library, CINAHL, and EMBASE.

These guidelines are inclusive and not prescriptive.

The recommendations were defined and graded based on the current levels of evidence and in accordance with the criteria adopted by American College of Chest Physicians. The recommendations were graded A, B, and C.

## Methodology

These guidelines were based on the last Italian Society of Colorectal Surgery (Società Italiana di Chirurgia Colorettale; SICCR) clinical practice guidelines for the evaluation and management of hemorrhoidal disease (HD) published in 2015 [1]. The goal of this consensus statement was to establish an evidence-based approach to HD.

The strategy Electronic sources such as MEDLINE, PubMed, Cochrane Review Library, CINAHL, and EMBASE were searched from March 1, 2009 to March 1, 2019.

Keywords combinations included were hemorrhoids, hemorrhoidal disease, internal and external hemorrhoids, thrombosed external hemorrhoids, sclerotherapy, rubber band ligation, Hemorrhoidal Laser Procedure (HeLP), Doppler-guided hemorrhoidopexy, Ferguson (closed) and Milligan–Morgan (open) hemorrhoidectomies, excisional hemorrhoidectomy, postoperative pain, anal stenosis, early and delayed bleeding, complications, special conditions, and minimally invasive procedures.

The criteria used to select evidence were study design [randomized-controlled trial (RCT), prospective and retrospective observational studies, case series, and systematic reviews], the presence of primary and secondary outcomes, study methodology (sampling, blinding, and analytical methods), English language, and the evaluation of papers published only in indexed journals with impact factor. Prospective randomized-controlled trials and meta-analyses were given preference in developing these guidelines.

Directed searches of the embedded references from the candidate articles were also performed.

The recommendations were defined and graded based on the current levels of evidence and in accordance with the criteria adopted by the American College of Chest Physicians (Table 1) [2].

**Table 1:** Grades of recommendation, assessment, development, and evaluation system grading recommendations

	Description	Benefit vs risk and burdens	Methodological quality of supporting evidence	Implications
1A	Strong recommendation, high-quality evidence	Benefits clearly outweigh risk and burdens or vice versa	RCTs without important limitations or overwhelming evidence from observational studies	Strong recommendation, can apply to most patients in most circumstances without reservation
1B	Strong recommendation, moderate-quality evidence	Benefits clearly outweigh risk and burdens or vice versa	RCTs with important limitations (inconsistent results, methodological flaws, indirect or imprecise) or exceptionally strong evidence from observational studies	Strong recommendation, can apply to most patients in most circumstances without reservation
1C	Strong recommendation, low- or very-low-quality evidence	Benefits clearly outweigh risk and burdens or vice versa	Observational studies or case series	Strong recommendation but may change when higher quality evidence becomes available
2A	Weak recommendation, high-quality evidence	Benefits closely balanced with risks and burdens	RCTs without important limitations or overwhelming evidence from observational studies	Weak recommendation, best action may differ depending on circumstances or patient or societal values
2B	Weak recommendations, moderate-quality evidence	Benefits closely balanced with risks and burdens	RCTs with important limitations (inconsistent results, methodological flaws, indirect or imprecise) or exceptionally strong evidence from observational studies	Weak recommendation, best action may differ depending on circumstances or patients’ or societal values
2C	Weak recommendation, low- or very-low-quality evidence	Uncertainty in the estimates of benefits, risks, and burden; benefits, risks, and burden may be closely balanced	Observational studies or case series	Very weak recommendations; other alternatives may be equally reasonable

*RCTs* Randomized-controlled trials

Three evidence levels were defined. The recommendations were graded A, B, and C.

Members of the SICCR were invited to contribute to the production of these guidelines and final recommendations were reviewed by the entire Clinical Practice Guidelines Committee. SICCR Clinical Practice Guidelines are updated every 4 years.

## Target users

The target users of guidelines are coloproctological surgeons, gastroenterologists, general practitioners, nurses, and other medical specialists who treat anoperineal diseases.

The guidelines may be used to inform clinical decisions and standards of care. The guidelines are also intended to inform patients about the possible alternatives for the management of their condition.

### Introduction: symptoms, classification, scoring system and diagnosis of HD

HD is the most common proctological disease with an estimated prevalence rate of 4.4%, with a peak in individuals between 45 and 65 years of age [3]. Furthermore, 50% of the population over 50 years old have experienced problems related to HD.

Symptoms of HD may overlap with those of other anorectal conditions such as skin tags, abscesses, fissures, polyps, inflammatory bowel disease (IBD), and anorectal neoplasms. The most common presentation of HD is painless rectal bleeding that occurs during or immediately after defecation. Usually, it is mild–moderate bright red bleeding which the patient observes on the feces or staining the toilet paper [4–7]. Recurrent bleeding may result in secondary iron deficiency anemia. Sometimes, HD may cause massive hemorrhage requiring urgent hospitalization and blood transfusions [8, 9]. Other symptoms to consider are swelling, prolapse, soiling, perianal skin irritation, itching, and discomfort. Furthermore, large hemorrhoidal prolapse may cause sense of rectal filling and, rarely, difficult defecation.

Pain is rare in case of uncomplicated HD. In fact, its presence may indicate other simultaneous painful conditions (fissure, abscess, pudendal neuropathy, proctalgia fugax, and anorectal neoplasm). Acute edema and thrombosis of external hemorrhoids (TEH) are responsible for acute anal pain irrespective of bowel movements. Although several HD symptom scores have been proposed so far [10–12], to date, none are widely used or considered the gold standard evaluation tool, even though after the publication of the HubBLe Trial [13, 14], the use of these scoring systems has increased to more easily compare data from the scientific literature.

### Classification and scoring system for HD

HD classifications should meet the need to choose the most suitable therapeutic approach as well as to have shared parameters for trials and guidelines.

Internal hemorrhoids are classified according to the presence and severity of prolapse as in the Goligher Classification [15].

Unfortunately, the Goligher classification has several limitations, because it does not consider the associated symptoms and their impact on quality of life, the etiopathogenesis of the disease, and specific clinical conditions such as circumferential prolapse or single prolapsed pile.

Therefore, it may not reflect the true severity of the disease and the effect of HD on the patient.

To overcome these limitations, different grading systems have been developed. All the grading systems are patient self-reported assessments focusing on the presence and frequency of different symptoms. Nystrom in 2009 used a five-point-based questionnaire assessing the frequency of pain, discomfort, itching, soiling, and need for manual reduction of hemorrhoids [11]. The system is easy to use and reproduce and has been successfully validated [13], but it fails to consider the presence and frequency of prolapse that does not need manual reduction. However, hemorrhoidal prolapse is a very important manifestation of HD and can impact on quality of life. Furthermore, the frequency of the symptoms was divided in four grades including “never”, “less than once a week”, “1–6 times per week”, and “every day”. Other grading systems assessing frequency of symptoms of hemorrhoids, similarly to those assessing severity of other conditions such as fecal incontinence, are based on five grades of frequency including” between never and less than once a week”. Yet, probably, the most important flaw of the Nystrom system is the lack of a score for the quality of life. HD is a benign condition and its severity is not only related to the frequency of its symptoms but rather to how they are perceived by the patient. Indeed, similar symptoms may affect patients’ life style and quality of life in very different ways with a significant variation from patient to patient. For this reason, quality of life should be considered when assessing the severity of HD. In 2011, Giordano et al. presented a similar system [16]. The severity of hemorrhoidal symptoms was scored using a specifically designed questionnaire assessing five different parameters each one scoring from 0 to 4 points, with zero indicating no symptoms and four daily symptoms or symptoms with every defecation. A score of zero corresponds to the complete absence of hemorrhoidal symptoms, while 20 corresponds to the worst possible symptoms. The five parameters assessed are bleeding, prolapse, need for manual reduction, pain, discomfort, and discharge as one parameter and impact on quality of life. This very simple and intuitive system has been proved useful and effective within clinical trials [17, 18]. Sodergren et al. [10] elaborated a more complex dedicated scoring system based on symptoms as reported by patients, taking into account how individual symptoms impact on patients’ quality of life. Based on their findings, the most relevant symptoms were selected and scored according to their frequency not in a linear way but according to what their expected impact on patients’ quality of life would be. Very surprisingly, the authors focused strictly on symptoms and the need for manual reduction was not considered. Yet, although strictly speaking this may not be a symptom, it is certainly an important sign of severity of HD and its frequency can impact on quality of life. While the work done by Sodergren and colleagues is very interesting and provides useful information about how this condition affects patients quality of life, it was validated on a small sample size *(n* = *45)* and it is not tailored to individual patients. It takes for granted that all patients suffering with HD are affected in the same way by each individual symptom, but this may not be necessarily always the case. Furthermore, because the score for each symptom is not linear, the system is not very easy to memorise and could be difficult to use in everyday practice.

Recently, Havard et al. [12] modified the Nystrom score considering how often the patient experiences prolapse instead of the need of manual reduction. Furthermore, they adapted the Short Health Scale (SHS), previously used in patients with inflammatory bowel disease (IBD) [19], to HD. This system remains very faithful to the Goligher classification while considering the quality of life using the SHS.

Apart from the previously mentioned system, some authors have proposed other classification [20–22] that are not widely used, due to their complexity.

### Diagnosis

**Diagnosis should focus on a related medical history for specific symptoms and risk factors corroborated by physical examination suggestive of HD*(Level of evidence: 1; Grade of recommendation: C).***

Diagnosis of HD should start with the collection of medical history identifying symptoms suggestive of the disease and risk factors such as constipation, a low fiber diet, sedentary lifestyle, and pregnancy. History of longstanding or uncontrolled portal hypertension should be considered for differentiate HD from anorectal varices [23]. Moreover, history of IBD and symptoms related to impaired anal continence should be investigated to plan the most appropriate treatment.

Physical examination should confirm the presence of HD ruling out other anorectal diseases. It should include inspection of the perianal tissues, anorectal digital examination, and the evaluation of hemorrhoidal prolapse degree during straining. The anorectal mucosa should be examined with an anoscope. The Sims position should be preferred because less embarrassing for the patient than prone position.

**Patients with HD and rectal bleeding should undergo colonoscopy to rule out other colorectal diseases *(Level of evidence: 1; Grade of recommendation: B)***

In Western countries, HD is one of the most frequent causes of severe acute lower gastrointestinal bleeding [8, 9]. Nevertheless, rectal bleeding is a common early symptom of colorectal cancer [24], as well as of other colorectal diseases such as IBD, diverticular disease, and angiodysplasia. For this reason, patients with rectal bleeding should undergo colonoscopy to rule out these diseases.

Colonoscopy should be mandatory in older patients and when there is a personal and/or a family history of colorectal neoplasms or documented advanced adenoma, IBD, history of altered bowel habits, recent significant weight loss, and a laboratory findings of iron deficiency anemia or a positive fecal immunochemical test (FIT) and guaiac-based fecal occult blood test (gFOBT) [25–30].

Flexible sigmoidoscopy may be associated with other screening modalities, such as gFOBT or FIT, in patients that are not willing or able to undergo colonoscopy [31, 32].

Sigmoidoscopy and colonoscopy should be integrated with anoscopy that has proven to have a higher detection rate of perianal pathology [33, 34].

Although an increased maximum resting anal pressure is a common finding in non-prolapsing hemorrhoids [35, 36], manometry is not routinely performed for diagnosis. Furthermore, anorectal endosonography is not usually indicated for the diagnosis of HD, but may disclose a thickening of submucosal tissue as well as of the internal or external anal sphincter [37].

### Conservative treatments

The goal of these treatments is the control of symptoms and not the correction of pathophysiological changes.

A balanced diet with adequate fiber and oral fluid intake may improve stool consistency and is one of the main purposes of lifestyle changes of the conservative treatment for HD. Constipation and in particular hard stool usually worsen symptoms related to the hemorrhoidal prolapse. A regular defecation with type 3 or 4 stool, according to the Bristol Stool Form Scale [38], without prolonged time on the toilet, to avoid straining during attempted defecation, may improve symptoms. Furthermore, the addition of anti-inflammatory agents or local steroids may be an effective first-line treatment and it should also be suggested as bridge to surgery. Local therapies such as anesthetics, antiseptics, and steroids show a temporarily relief of HD-related symptoms, but the efficacy of their prolonged application is not demonstrated and could induce local reactions or sensitization [39, 40].

### Fiber and/or laxatives

**Daily oral intake of fiber, either food or supplements, shows a consistent beneficial effect for HD symptoms reducing the risk of bleeding, in case of an acute event, and as the risk of not improving symptoms in about 50% and 47% of patients, respectively. Several trials show a lack of evidence regarding a direct effect on prolapse, pain and itching *(Level of evidence: 1; Grade of recommendation: B).***

Dietary fiber intake is generally used in patients with I–II-degree HD even if it can be effective in more advanced stages. Fiber restores the normal frequency of bowel movement thanks to the increase in fecal mass, volume, and softness. Fiber should be associated with an adequate oral fluid intake, although its efficacy in treating constipation remains controversial [41, 42]

Stimulant laxatives or osmotic agents have been shown to be effective for the treatment of HD symptoms in several randomized trials with consistent results over time in reducing the risk of bleeding as well as the risk of persisting symptoms if compared with the placebo group [43]. However, the methodology was often too weak to draw the final conclusions and more attention needs to be given to the cost-effectiveness ratio.

### Sitz bath

A sitz bath with warm water (not exceeding 40–42 °C for 3 min) is a traditional and frequently recommended remedy for a variety of anal disorders including HD [44]. Unfortunately, the proper instructions to execute it are rarely given to patients [45]**.**

There is a lack of RCTs defining the role of sitz bath with warm water in the treatment of HD-related pain ***(Level of evidence 2; Grade of recommendation C).***

Pain relief may be related to internal sphincter relaxation with a decrease of anal resting pressure [46] according to the thermosphincteric reflex described by Shafik in 1993 [47]. Another option is to sit upon a warm water bag to avoid the vacuum below the buttocks.

Despite its benefit, it can be difficult for the patients to perform in the hospital or at home [48].

For this reason, another option is to sit upon a warm water bag. Furthermore, Hsu et al. demonstrated that the warm water spray method can be a safe and easily performed alternative to size bath [49]

### Phlebotonics

**Phlebotonics has a statistically significant effect on HD-related symptoms (bleeding, pain, itching, and symptoms recurrence) if compared with a control group **[50, 51] ***(Level of evidence: 1; Grade of recommendation: B).***

Phlebotonics are a heterogeneous class of drugs composed by products extracted directly from plants such as flavonoids or synthetic compounds as calcium dobesilate. They simultaneously increase the vascular tone and the lymphatic drainage, decreasing vascular capacity, and stabilizing capillary permeability. However, their precise mechanism of action is not completely understood [50].

They are usually well tolerated with a few adverse effects. Their main side effects are mild symptoms as headache, gastrointestinal symptoms, or tingling sensations [52].

Furthermore, a prolonged exposition to high levels of flavonoids (many times more than their common dietary sources), through an unbalanced diet or by supplementation, may lead to an excess of reactive oxygen species formation and subsequent deoxyribonucleic acid (DNA) damage. These effects may be relevant during pregnancy, because flavonoids can cross the placenta [53].

A meta-analysis [54] including 14 trials and 1514 patients found that the use of flavonoids decreases the risk of worsening or persisting symptoms by 58% [relative risk (RR) 0.42 (95% confidence interval (CI) 0.28–0.61)] and showed an apparent reduction in the risk of bleeding [RR 0.33 (95% CI 0.19–0.57)], persistent pain [RR 0.35 (95% CI 0.18–0.69)], itching [RR 0.65 (95% CI 0.44–0.97)], and recurrence [RR 0.53 (95% CI 0.41–0.69)]. However, limitations in the quality and heterogeneity of the trials examined make this conclusion unreliable leaving open the question about the real efficacy of phlebotonics.

### Traditional Chinese medicine

Traditional Chinese medicine is based on the use of medicinal herbs.

In the nine published trials included in a Cochrane review, the herbs are divided into two types: patent herbal medicine or the self-produced compound. The most frequent herbs used are: Radix Sanguisorbae, Radix Rehmanniae, Fructus Sophorae, Radix Angelicae Sinensis, Radix Scutellariae, and Cacumen Biotae. Their dosage is rarely reported resulting in a huge bias which limits the study reproducibility [55].

Traditional Chinese herbs were not proved as useful for stopping bleeding from hemorrhoids in a Cochrane Review [55] ***(Level of evidence: I; Grade of recommendation: B).***

### Outpatient treatments

#### Rubber band ligation (RBL)

**RBL is the most popular non-invasive procedure **[56]** and should be used for the treatment of I, II, and III-degree HD that fails conservative treatment*(Level of evidence: 1; Grade of recommendation: B).***

A rubber band is applied to the base of the internal hemorrhoid, above the dentate line to avoid severe pain, causing ischemic necrosis, fibrosis, and fixation of the remaining mucosa. Usually, the necrotic hemorrhoidal tissue drops out within the following 7–10 days.

According to a recent Italian survey of over 32,000 patients, II-degree HD is treated with RBL in over 90% of the patients [57]. RBL is contraindicated in patients on anticoagulants or with a bleeding disorder, thrombosed hemorrhoids, concomitant anorectal sepsis, anal fissures, abscess and fistula, colitis, colorectal tumors, pregnancy, immunodeficiency, and diabetes mellitus.

Although the procedure is often avoided in patients with anticoagulants, according to one of the largest retrospective studies regarding RBL, only 2.9% of the patients taking warfarin or anti-inflammatory drugs bled post-procedure [58]. These results were confirmed by Hite al [59], who demonstrated that Clopidogrel does not increase bleeding complications in the postoperative period.

A recent cost-effectiveness analysis [60] of 2026 patients undergoing RBL for symptomatic HD with six board-certified colorectal surgeons between March 2012 and March 2017 stated that RBL had a lower average estimated cost and a lower average quality-of-life deficit per patient if compared with hemorrhoidal artery ligation (HAL), stapled hemorrhoidopexy (SH), or surgical hemorrhoidectomy. In this review, only 6% of the entire cohort required surgical treatment; meanwhile, most of the patients solved the problem with further banding procedures. In fact, repeated RBL treatment were reported in 8–10% of patients, with a recommended 4-week interval between the sessions [14, 61-68]

In the HubBLe trial [14], 185 patients were assigned to the HAL group and 187 to the RBL group. Patients treated with RBL had a lower rate of pain (1 versus 5) and bleeding requiring transfusion (1 versus 2) but a higher rate of recurrence. However, patients may prefer RBL as the first-line treatment.

A Dutch national survey demonstrated the superiority of RBL for II-degree HD and of excisional procedures for III-degree HD [69].

In RBL, bleeding stops in up to 90% and improvement in II-degree HD has been shown in 93–100% of patients (63, 64, 70). Furthermore, III-degree HD improves in 78–83.8%, but IV-degree prolapse should have a more invasive treatment [70, 71].

The possible minor complications of the technique include pain, bleeding, thrombosis, and skin tags [70].

Unfortunately, rare but severe complications such as massive gastrointestinal hemorrhage [72], liver abscesses [73], endocarditis [74], perineal sepsis, and also death [75] were described after RBL.

### Sclerotherapy

**Injection Sclerotherapy (IS) should be used for the treatment of I–II and III-degree HD that fail conservative treatment *(Level of evidence: 1; Grade of recommendation: B)***

IS, initially described by  Morgan in England in 1869 [76], is the injection of sclerosing agents at the apex of the internal hemorrhoidal complex, above the dentate line, leading to moderate tissue destruction with scarring, fibrosis, and fixation of the hemorrhoidal tissue. Several sclerosing agents have been described and used [5% phenol in almond oil, aluminum potassium sulfate and tannic acid (ALTA), and 50% dextrose water].

Among these agents, ALTA seems to be the most effective one, even if in low resource countries, 50% dextrose water could be a safe and effective alternative [77-85].

Moser et al. [79] in 2007 introduced foam sclerotherapy with polidocanol 3%. Subsequently, the authors compared, in a randomized, controlled, single-blind, multicenter trial, polidocanol foam with liquid polidocanol in the treatment of I-degree symptomatic HD demonstrating the superiority of the foam, after 12-week-follow-up, regarding success rate after one IS session (58/66 pts 88% vs 44/64 pts 69%; *p* = 0.01), number of session required for success [1.08 (± 0.32) vs 1.42 (± 0.64); *p* < 0.001), and total amount of injected polidocanol (35 mg (± 10) vs 85 mg (± 38); *p* < 0.001). Only one adverse drug reaction (acute prostatitis) was observed in the foam group. After that complication, the authors modified the injection technique placing the first injection at 11 o’clock.

Probably, the low dose of drugs used for the foam injection will lead to a decrease of the complication rate.

Several studies reported a 92–100% improvement in bleeding of patients with II- and III-degree HD with the use of IS [70, 77, 81, 83]. Resolution of prolapse was reported in 85–94% of patients with II–III-degree hemorrhoids with 5 year follow-up [70, 77, 81].

Subjective moderate/excellent improvement was reported in 70–92% of patients suffering from III- and IV-degree HD [81, 84]. Recurrence of prolapse is currently 15% at 1 year in unselected II degree [81, 86]; meanwhile, the failure rate at 1 year was reported to be, respectively, 25% and 80% in III-degree HD patients treated with ALTA and 5% phenol in almond oil [81, 82].

Patients reported a relatively low rate of postoperative pain (24–49%) [79, 86]: the intra-procedural injection was painful in up to 90% [81]. Bleeding is rare.

Mucosal ulceration is one of the most frequent complications, reported in 3.6% of patients [81]. Major complications including impotence, severe acute liver injury, fistula formation [87], fatal necrotizing fascitis, and abdominal compartment syndrome following sclerotherapy have been reported [88-91].

### Infrared coagulation

Infrared Coagulation (IRC) should be used for the treatment of I-II and III-degree HD that fail conservative treatment ***(Level of evidence: 1; Grade of recommendation: B).***

It consists of the exposure of the internal hemorrhoids to infrared waves, resulting in a protein coagulation and necrosis, immediately visible as a white spot.

Dimitrolopoulus et al. [92] reported a success rate of 78%, 51%, and 22%, respectively, for I-, II-, and III-degree HD with a cumulative subjective improvement of 81–93% for I–II-degree HD [93, 94].

The most frequent complication of IRC is a post-procedural pain which occurs in 16–100% of patients [92, 95]. The incidence of postoperative bleeding is 15–44% [92, 95].

Recurrence of bleeding is reported in 13% of patients at 3-month follow-up [92, 95]. Data are insufficient for the assessment of the long-term efficacy of the technique.

### Non-excisional procedures

#### Stapled hemorrhoidopexy

**SH is an effective technique for the treatment of HD. When compared with conventional hemorrhoidectomy, SH is associated with less operating time, earlier return of bowel function, shorter hospital stay, less pain, a faster functional recovery with shorter time off work, an earlier return to normal activities, and better wound healing (*Level of evidence 1; Grade of recommendation: A*).**

**However, the incidence of recurrence and the need for additional operations are also significantly higher when compared to conventional hemorrhoidectomy (*Level of evidence 1; Grade of recommendation: A*).**

Numerous studies on short-term outcomes have demonstrated that when compared to conventional hemorrhoidectomy, SH is quicker to perform and patients experience less postoperative pain, postoperative bleeding, wound complications, and constipation [96-101]. Hospital stay and time to return to normal activities were also shorter. Furthermore, the requirements for non-surgical and surgical reinterventions and the readmission rate are similar following SH and conventional hemorrhoidectomy [99]. However, meta-analyses looking at long-term outcomes after SH and conventional hemorrhoidectomy found significantly higher recurrence rates following SH [99-101].

The higher recurrence rate was confirmed by a recently published retrospective study that analyzed the long-term outcome (15-year follow-up), of 257 patients who underwent SH [102]. Follow-up data were available in 140 cases even if only 116 answered the questionnaire regarding recurrence. 55 patients reported the recurrence of at least one hemorrhoidal symptom and 17 patients required a further surgical treatment. Large capacity stapling devices may lead to better results, but this remains unclear [103, 104].

SH is more expensive than traditional excisional surgery. The cost–utility analysis indicates that SH has < 0.1% probability of being cost-effective at £20,000 and 0.1% probability of being cost-effective at a £30,000 willingness to pay threshold [105] ***(Level of evidence: 1; Grade of recommendation: A).***

Although all major prospective randomised trials have failed to demonstrate any significant adverse event related to the use of SH, in up to 10% of these patients, several complications were observed [99]. Numerous minor and major complications have been widely reported outside the major trials [106, 107] ***(Level of evidence: 2; Grade of recommendation: C).***

**Transanal hemorrhoidal dearterialization (THD) or Doppler-guided hemorrhoidal artery ligation (DGHAL)**

**THD or DGHAL is a treatment option for II- and III-degree haemorrhoids and in experienced hands possibly also for IV degree **[18, 108, 109] ***(Level of evidence: 1; Grade of recommendation: A).***

**THD/DGHAL is associated with decreased postoperative pain, reduced postoperative events, and faster recovery than excisional hemorrhoidectomy, but carries higher recurrence rates*** (****Level of evidence: 1; Grade of recommendation: A).***

Following THD, fewer patients had postoperative bleeding compared with open hemorrhoidectomy or SH. THD is associated with fewer emergency reoperations than open, closed, stapled and LigaSure™ procedures, with a high probability of being the best treatment regarding this outcome (*p* = 0.710) [110]. In addition, it has been shown that compared to more invasive surgical techniques THD is associated with shorter operating time, less postoperative complications, and notably decreased postoperative pain. This resulted in a shorter length of hospital stay and an earlier time to the first bowel movement [110]. Work or normal daily activities were also resumed quicker following THD (*p* = 0.09) [111]. However, Other studies demonstrated that compared with hemorrhoidectomy, dearterialization with mucopexy resulted in similar postoperative pain and morbidity and a similar 24-month cure rate [112, 113] ***(Level of evidence: 2; Grade of recommendation: B).***

Three studies comparing the use of Doppler to a blinded transfixation suggested that operative time was significantly longer and the postoperative pain score higher with the use of Doppler, while there was no difference in recurrence rates [114-116]. However, bias regarding technique and instrumentation used in these studies make their results difficult to interpret and as such the quality of the data is not good enough for a recommendation.

When compared to SH, THD was associated with significantly less postoperative pain. Both techniques were equally effective in the short term with similar rates of complications and recurrence [16, 117-120] ***(Level of evidence: 1; Grade of recommendation: B).***

The recurrence rate following THD/HAL seems to be influenced by surgical experience. Overall recurrence rate ranges between 3 and 24% (HAL 3.3–24%; THD 3–20%) with reintervention, to treat recurrent symptoms, necessary in 2.7–22% of patients (HAL 2.7–22%; THD 4.1–17.8%) [17].

The use of a mucopexy has become a routine part of the procedure though, in patients with bleeding as the only symptom, dearterialization alone may suffice. In the presence of any degree of hemorrhoidal or mucosal prolapse, mucopexy should be added to the dearterialization. A THD technique with targeted mucopexy has been described as the best way to tailor this procedure to each individual patient [58].

Pain following THD can occur in up to 38% of operated patients (HAL range 0–38% of patients; THD range 0–35% of patients). Yet, in the majority of series, the incidence of postoperative pain was less than 10%. Postoperative bleeding was reported in up to 18% of patients (HAL range 0.9–18%; THD range 0–13%) and, in rare instances, required hospital admission and reintervention. Other postoperative events include tenesmus, which is more frequent in patients who underwent mucopexy, hemorrhoidal thrombosis, observed in up to 8.6% of patients (HAL range 2.3–6.7%; THD range 0–8.6%), and anal fissure (2.1% of patients; HAL range 0.9–2.1%; THD range 0.6–1.5%). Transient fecal urgency has also been reported [17].

### Excisional procedures

#### Excisional hemorrhoidectomy

The traditional excisional methods (Milligan-Morgan, Ferguson procedures) still remain the first choice and the most common indication for symptomatic III- and IV-degree HD ***(Level of evidence: 1; Grade of recommendation: A)****.*

Open hemorrhoidectomy (OH) and closed hemorrhoidectomy (CH) are both efficient surgical procedures for the treatment of HD, despite the presence of some disadvantages due to the extent of dissection as well as to the presence of wounds below the dentate line with postoperative pain that can be severe delaying the return to normal daily activities. According to a recent meta-analysis [121] of 11 RCTs and 1326 patients comparing OH and CH, the Ferguson procedure was associated with reduced postoperative pain, faster wound healing, lesser risk of postoperative bleeding, and longer procedure time ***(Level of evidence: 1; Grade of recommendation: A).***

Radiofrequency hemorrhoidectomy is a sutureless technique dependent on a modified electrosurgical unit to achieve tissue and vessel sealing. It results in less blood loss, postoperative pain, and complications. It is technically simple, because suturing is not required and hemostasis is easy to achieve [122]. It has the potential of making hemorrhoidectomy into a day-care regimen ***(Level of evidence: 1; Grade of recommendation: B)****.*

Pain following hemorrhoidectomy is well described in the literature and seems to be less after radiofrequency hemorrhoidectomy [123-125]. Postoperative bleeding is reported in up to 3% of patients (OH range 0.2–5%; CH range 0–4%). The overall recurrence rate is between 2 and 8% (OH 2.8–7.8%; CH 2–8%). Fecal incontinence following hemorrhoidectomy is reported as 6% with no significant difference between OH and CH [126]. There is no difference between OH and CH  regarding the rate of fecal impaction, anal stenosis, anal fissure, and some loss of the sensitive anal mucosa [110].

### Management of HD in special conditions

#### Pregnancy

The prevalence of symptomatic hemorrhoids is higher in pregnant than in non-pregnant women.

Pregnancy and spontaneous vaginal delivery are well-established predisposing factors for the development of HD in females due to the increased intra-abdominal pressure from uterine growth, the hormonal changes, and constipation (38% of the pregnant females). Clinical reports demonstrated that HD is mostly prevalent in the last trimester of pregnancy and in the first month after delivery, with about 25–35% of the pregnant females suffering from this disease [127, 128]. In particular, thrombosed external hemorrhoids (TEH) are more frequent during the last trimester of pregnancy and immediately after delivery (7.8% and 20%, respectively). The prevalence of symptomatic hemorrhoids in pregnant females is higher with the increase in age and parity [129, 130]. Insufficient data exist on the safety of anti-hemorrhoidal treatment in pregnancy.

Patients with I- and II-degree HD may benefit from oral rutosides for symptom relief. However, their use cannot be recommended until new evidence about their safety is available [131] ***(Level of evidence: 1; Grade of recommendation: B)****.*

Sitz baths have been shown to be a statistically significant choice in achieving a complete healing of HD in pregnant females compared to conservative treatment with an anorectal cream (*p* < 0.005) [132–134] ***(Level of evidence: 2; Grade of recommendation: C)****.*

Although there is a tendency toward conservative treatment, hemorrhoidectomy (CH) has been successfully performed without risk to the fetus [134]. In fact, excisional surgery should be considered, especially in case of hypotensive risk due to postoperative bleeding. In this case, excision of the symptomatic pile is required ***(Level of evidence: 2; Grade of recommendation: C)****.*

According to Mirhaidari et al. [135], an excision under local anesthesia in an outpatient regimen of the thrombosed pile/s can be easily performed without any special monitoring as well as any risk of preterm labor or miscarriage ***(Level of evidence: 2; Grade of recommendation: C)****.*

There are no safety data available for any of the compounds commonly used for HD during pregnancy. Thus, it should be treated by increasing fiber and oral fluid intake, administering stool softeners, improving toilet habits, and sometimes by adding topical treatment [132].

The course of HD tends to be longer during pregnancy and most symptoms will resolve spontaneously after delivery, with a few cases requiring a surgical evaluation during pregnancy or after delivery.

#### Thrombosed hemorrhoids

TEH can be easily recognized on physical examination as usually tender visible blue perianal/anal lumps. TEH most frequently causes acute and severe pain, but the severity of the symptoms depends on the size of the thrombus. Without intervention, the pain typically gets better over 2–3 days, with a continuous improvement as the thrombus gradually absorbs over several weeks. Analgesics and stool softeners may be beneficial.

Heparin treatment [136], a highly standardized and bioavailable mixture of flavonoids and triterpenes [137], topical nifedipine [138], and botulinum toxin injection [139] are reliable options especially in delaying or avoiding a surgical procedure.

Conservative treatment for prolapsed thrombosed internal haemorrhoids, if compared with urgent hemorrhoidectomy, is associated with a shorter inpatient stay and less anal sphincter damage than operative treatment [140] ***(Level of evidence: 1; Grade of recommendation: B)****.*

Excision has been shown to have better results in terms of reduction of pain, recurrences, and number of skin tags in comparison to simple incision and conservative treatment with glyceryl trinitrate (GTN) (*p* < 0.001) [141] ***(Level of evidence: 1; Grade of recommendation: B)****.*

A literature search [142] considering 800 articles on hemorrhoids stated that excision allows better results than incision or topical GTN meanwhile symptoms last over 3 weeks with conservative treatment ***(Level of evidence: 1; Grade of recommendation: B)***. This latter can be avoided by combining topical nifedipine and lignocaine rather than using lignocaine alone ***(Level of evidence: 1; Grade of recommendation: B)****.*

Thus, most patients with TEH benefit from surgical excision within 72 h of the onset of symptoms [143] ***(Level of evidence: 1; Grade of recommendation: B)****.*

These data were confirmed by Jongen and Coll [144] who conducted a retrospective analysis of complication rates, symptom recurrences, long-term results, and patient satisfaction after outpatient excision (local anesthesia) of TEH in 340 patients. They concluded that outpatient excision of TEH under local anesthesia can be safely performed with a low recurrence and complication rate and with a high level of patient acceptance and satisfaction ***(Level of evidence: 2; Grade of recommendation: C)****.*

Zuber [145] proposed hemorrhoidectomy for TEH. He suggested that hemorrhoidectomy be performed through an elliptic incision over the site of thrombosis with removal of the entire diseased hemorrhoidal plexus in one piece. He underlined that caution must be exercised to avoid cutting into the muscle sphincter below the hemorrhoidal vessels. Infection after suture closure is rare secondary to the rich vascular network in the anal area. Stool softeners must be prescribed postoperatively to help prevent tearing at the suture line. Moreover, training and experience in general and skin surgery are necessary before the physician attempts this procedure unsupervised.

SH is a feasible treatment for selected patients with an acute hemorrhoidal crisis and has a similar complication rate if compared with a conventional excisional hemorrhoidectomy. SH is associated with less postoperative pain, shorter operation time, a shorter hospital stay, and an earlier return to normal activities [146] ***(Level of evidence: II; Grade of recommendation: B)****.*

However, older patients with anemia or a prolonged hemorrhoidal crisis are unsuitable for stapled hemorrhoidectomy [147] ***(Level of evidence: II; Grade of recommendation: C)****.*

#### Immunosuppressed patients

HD is common in patients with acquired immunodeficiency syndrome [AIDS], often resulting from chronic diarrhea brought on by medications.

As suggested by Gupta [148], selective management will result in high rates of symptomatic relief and complete wound healing after HD surgery without excessive morbidity and mortality.

Even if the indications for hemorrhoidectomy in patients with AIDS need to be considered extremely carefully because of the high incidence of delayed wound healing [149], nowadays, there is no significant increase in complication rate for patients with a low CD4 + T-cell count (< 200/μL) compared to those with a higher count [150] ***(Level of evidence: I; Grade of recommendation: C)****.*

Recently, Fan and Coll [151] reported that tissue-selecting therapy stapler (TST) for prolapsing hemorrhoids in human immunodeficiency virus (HIV)-infected patients is a safe technique with a low complication rate and minor technical difficulties, especially for HIV-infected patients who have a high satisfaction index ***(Level of evidence: 2; Grade of recommendation: C)****.*

In conclusion, there is no evidence for the best treatment regarding HIV + patients with hemorrhoids. Further studies are requested to provide some scientific evidence. Moreover, no data of transplanted patients have been reported in the international literature.

####  Inflammatory Bowel Disease

There is no consensus in the scientific literature regarding the exact indications for surgery in patients with IBD who have HD.

D’Ugo et al. [152] suggested that first-line management should be medical therapy, considering that a spontaneous healing is possible. Despite the higher risk of complications in patients with IBD [153], in non-responding patients, the surgical options on a highly selective basis can be considered with acceptable results [154] ***(Level of evidence: 2; Grade of recommendation: C)****.*

#### Radiation proctitis

There is no consensus in the scientific literature regarding the exact indications for surgery in patients who have had pelvic radiotherapy for malignancy.

Thornhill et al. [155] reported severe complications in the treatment of HD in patients with radiation proctitis ***(Level of evidence: 2; Grade of recommendation: C)****.*

After radiation in the pelvic region, most symptoms are linked to the radiotherapy and not to the HD. For this reason, any invasive procedure for benign disease, especially for hemorrhoidal-like symptoms, must be strongly discouraged [156].

#### Coagulopathies

The frequent use of anticoagulants has likely led to an increased incidence of bleeding in patients with a clinically significant internal hemorrhoids. Society guidelines recommend that anticoagulation be suspended prior to hemorrhoidal surgery and procedures.

However, there is no consensus regarding the exact indications for surgery in patients with HD affected by coagulopathy.

As already described, Hite et al. [59] reported that the risk of a bleeding complication after RBL for  HD does not appear to be increased in patients taking clopidogrel ***(Level of evidence: 1; Grade of recommendation: C)****.* These results were confirmed by Atallah et al. [157] who reveal that THD can be performed on anticoagulated patients without cessation of oral agents without an increased risk of postoperative bleeding. Nevertheless, Albuquerque [158] suggested that secondary bleeding normally occurs 10–14 days after RBL and patients taking anti-platelet and/or anti-coagulant medication have a higher risk, with some reports of massive life-threatening hemorrhage ***(Level of evidence: 2; Grade of recommendation: C)****.*

### Emerging technologies

#### Embolization of the hemorrhoidal arteries

Embolization of the hemorrhoidal arteries, the so-called “Embhorroid technique”, was first described for the treatment of HD in 2014 by Vidal et al. [159].

It consists in the embolization of the hemorrhoidal arteries, in which arterial occlusion is achieved through an endovascular approach (usually transfemoral) using coils placed in the terminal branches of the superior rectal arteries. The use of polyvinyl alcohol (PVA) particles of 0.3 mm and metallic coils seems to be more effective in symptom relief than the use of metallic coils alone. In fact, the use of 0.3 mm particles determines a more distal hemorrhoidal plexus embolization reducing the reloads by the middle rectal arteries and avoiding rectal ischemia, because the particles do not pass the inferior rectal artery anastomoses [160].

It could be performed in an outpatient setting and has been shown to be a safe and effective technique for the treatment of II–III-degree HD [161] ***(Level of evidence: 2; Grade of recommendation: C)****.*

It should be reserved for a selected group of patients with disabling and refractory hemorrhoidal symptoms and without irreducible prolapse [162] ***(Level of evidence: 2; Grade of recommendation: C)****.*

Emborrhoid showed good bleeding control in patients with contraindications for conventional surgery with a clinical score improvement in 72% of cases after the first embolization session [162] ***(Level of evidence: 1; Grade of recommendation: C)****.*

#### HeLP

HeLP is the acronym for Hemorrhoidal Laser Procedure and is based on the application of a 980-nm diode causing shrinkage of the terminal branches of the superior hemorrhoidal artery [163, 164].

To carefully detect the superficial arteries at approximately 2.5 cm above the dentate line, a 20 MHz Doppler transducer is used. The laser energy delivered at 980 nm wavelength at that level induces a shrinkage up to a depth of 4 mm, thus reducing the blood flow [165].

HeLP has been shown to be safe, effective, and easy to perform. It is an effective alternative for the treatment of symptomatic hemorrhoids, especially with bleeding and pain as prominent symptoms, in the absence of severe mucosal prolapse even if an improvement of the latter has been described. This procedure can be also associate with rectoanal repair or with mucopexy ***(Level of evidence: 1; Grade of recommendation: C)****.*

This novel technique shares the rationale of the HAL and THD procedures but with the potential advantage of being less invasive and not requiring general anesthesia [166] ***(Level of evidence: 1; Grade of recommendation: C)****.*

The most common intraoperative procedure-related complication reported is postoperative bleeding, ranging from 5.9% to 8.8% of cases with more than a half of them needing an hemostatic procedure [164, 166] ***(Level of evidence: 2; Grade of recommendation: C)****.*

Short- and long-term postoperative complications rate is very low with pain as the most significant postoperative symptom. It required medications for an average of 3 days after surgery in around 9.5% of patients [166]. Anyway, it has also been reported a case of life-threatening condition in which it was necessary to fashion a diverting stoma due to bowel obstruction after a postoperative rectal hematoma [167].

Symptoms recurrence ranges from 10 to 20% for II–III-degree HD [166, 168] ***(Level of evidence: 2; Grade of recommendation: C)****.*

### Laser hemorrhoidoplasty

Laser hemorrhoidoplasty (LHP) is based on the application of the laser beam inside the hemorrhoidal tissue. After making a 1-mm opening at the cutaneous anal edge of the hemorrhoidal pile, the fiber is introduced inside the tissue parallel to the anal sphincter as well as to the rectal axis. The fiber is then pushed up to the upper part of the piles and three pulses at a power of 15 W are delivered. This maneuver is repeated thorough the same hole but in different directions. The laser beam induced a shrinkage of underlying tissues up to approximately 5 mm of depth [169].

LHP seems to reduce postoperative pain, intraoperative bleeding, and the need of postoperative analgesics if compared with Milligan–Morgan procedure, with a complete resolution of symptoms in about 70% of cases [169] ***(Level of evidence: 2; Grade of recommendation: B)****.*

It is associated with shorter operative time and less postoperative pain compared to excisional surgery [170]. Furthermore, in a recent observational study concerning 50 patients with II- and III-degree HD, Brusciano et al. [171] reported a quick return to daily activity 1 day (40%) and 2 days (100%) after the procedures. ***(Level of evidence: 2; Grade of recommendation: C)****.*

After a mean follow-up of 5.4 years, recurrences was reported in 39% and 33% of patients with II- and III-degree HD, respectively [172], without any statistically significant differences related to the degree of HD (*p* = 0.761) ***(Level of evidence: 1; Grade of recommendation: C)****.*

### Complications of surgical treatments for HD

1. Open and closed hemorrhoidectomies have a significantly more severe negative impact in the early postoperative period than stapled, THD, LigaSure™, and Harmonic™ hemorrhoidectomies ***(Level of evidence: 1; Grade of recommendation: B)***

Various studies have shown that the closed and radiofrequency hemorrhoidectomies  had significantly more postoperative complications (mainly pain) than the open, stapled, LigaSure™, Harmonic™, and THD  procedures. Furthermore, OH and CH were associated with greater operative blood loss and a longer operating time compared with the other surgical techniques. Nevertheless, a low recurrence rate is perceived to be the most important advantage of OH and CH which were found to have a lower recurrence rate than THD and SH. Moreover, the use of energy devices such as LigaSure or Harmonic may reduce complication rate, even if with increased costs [110]. Overall procedural complication rates of SH ranged from 2 to 68%. However, these complications may typically occur in about 16% of procedures [147, 173, 174].

The overall complication rates after SH and THD were comparable with no significant differences [175, 176]. Despite a more favourable postoperative period for SH or THD/DGHAL techniques, some procedure-specific complications have been described, and should be considered during preoperative discussion with the patients regarding indications for surgery.

Early fecal urgency after SH has been reported, with incidence rates of 0—25%, and a median of 8% [173].

On the other side, postoperative tenesmus was reported in up to 24% of patients and pruritus in up to 15% after DGHAL, especially if mucopexy was contemporary performed [177].

Several studies described different complication rates following office procedures (such as RBL, sclerotherapy, and infrared coagulation), ranging from 3% to 18.8% [158, 178]. A review of 39 studies including 8060 patients who had RBL revealed post-banding complications in 14% of the patients, although severe complications are rarely reported [179].

Urinary retention is one of the most common complications after surgery for  HD, with incidence rates of 3–50% with most studies reporting a rate around 15% [180, 181].

2. Emergency reoperation may be required in about 2% of patients after a surgical treatment for HD ***(Level of evidence: 1; Grade of recommendation: B)***

Up to 90% of emergency reoperations are needed to stop a postoperative bleeding. Interestingly, most patients will not have an identifiable source of bleeding by the time which they are examined in the operating room. However, these bleeding episodes can be significant and a return to the operating room for a second look may be justified. Intractable pain, hematoma incision, residual hemorrhoidal thrombosis, and sepsis are other possible indications for reoperation.

A network meta-analysis of the trials reporting on reoperation showed that THD/DGHALprocedures were associated with significantly fewer reoperations than open, closed, stapled, and LigaSure™ procedures, in large part due to lower bleeding rate [110]. However, THD had a higher recurrence rate than open, closed, LigaSure, laser, and radiofrequency hemorrhoidectomy, and therefore, the highest probability of being the worst treatments regards recurrence of hemorrhoids.

3. Bleeding: fewer people have postoperative bleeding after THD/DGHAL procedures compared with OH or SH ***(Level of evidence: 2; Grade of recommendation: B)***

For conventional hemorrhoidectomy (Milligan–Morgan and Ferguson) and bipolar energy device hemorrhoidectomy (LigaSure), rates of bleeding between 0 and 49% have been reported. Clinically significant bleeding has been reported in 0.3–6% of patients, with an average of around 2%, and need of reintervention between 1–2%, without a significant difference in rates of bleeding between the procedures [121, 182, 183].

Early bleeding was the most common complication after SH, with the overall rate following the procedure ranging from 0 to 68% (median 7.5%) with < 1% of postoperative bleeding requiring treatment [176, 184].

Bleeding after Doppler-guided hemorrhoidal dearterialization has been reported to be low (0–22%, median 4.3%); however, this needs to be balanced with the chance of long-term recurrence [108].

Bleeding after RBL normally occurs between 5 and 14 days after treatment, probably due to the sloughing of the ligated hemorrhoids [182].

However, when RBL was compared to HAL, recurrence rates (if RBL was repeated), symptom scores, complications (such as postoperative bleeding), quality-of-life score, and continence score were similar, although patients had more pain in the early postoperative period after HAL. HAL was also more expensive and was not found to be cost-effective compared with RBL in terms of incremental cost per quality-adjusted life-year [14].

4. Pain: The higher levels of pain related to OH and SH compared to other techniques resulted in a longer hospital stay and a later return to normal activities. A multimodal pain reliever regimen should be used to promote a faster recovery, prevent urinary retention, and improve patient satisfaction*** (Level of evidence: 1; Grade of recommendation: B)***

Compared to excisional hemorrhoidectomy, THD and SH are followed by less postoperative pain. A number of modifications in surgical and postoperative management have been proposed and attempted to reduce the pain, with variable results.

Topical 2% Diltiazem or GTN ointment demonstrated a significant pain reduction in randomized trials [185].

Lateral sphincterotomy or botulin toxin injection also demonstrated efficacy in reducing postoperative pain, suggesting a possible role of sphincteric spasm in its pathogenesis. However, the risk of developing temporary or permanent anal continence alterations limits the use of sphincterotomy.

The use of oral metronidazole in controlling postoperative pain was recently evaluated in two meta-analysis with conflicting results [186, 187].

Several other treatments such as mesoglycan [188] were recently used for pain after hemorrhoidectomy, but further trials are needed to reach agreement.

The reported incidence of postoperative pain ranged from 0 to 38% with a pooled value of 15% after THD procedure [175].

Pain after SH has been attributed to the involvement of the staple line on the sensitive squamous epithelium of the anoderm, inclusion of smooth muscle, or surrounding anorectal tissue in the scar, and induction by the staple line of an inflammatory response in the rectal ampulla, sphincter or rectal spasm, elevated anal resting pressures, retained staples, fibrosis around the staple line, wound dehiscence, and sepsis. Although chronic pain after SH has been variably reported, it is typically experienced by less than 2% of patients.

Treatment of chronic pain following HD surgery should be targeted to the underlying source. However, it is usually quite difficult to manage and cure, which emphasizes the importance of proper knowledge of the anatomy and careful use of surgical techniques.

Warm sitz baths and non-steroidals therapies can relieve mild pain. Antispasmodics such diazepam or cyclobenzaprine may be added if levator spasm is noted. Post evacuatory pain may be treated with oral nifedipine. Anismus may be treated with botulinum toxin injection. For selected cases, sacral neuromodulation has also been described [189].

In case of chronic pelvic pain after stapler surgery, the removal of staples or staple line excision has been reported [106, 190]. However, the evidence of these treatments is low and effectiveness observed only in a low percentage of patients.

Urinary retention after hemorrhoidectomy is often multifactorial, with pain as one of the major issues, causing symptoms through irritation of pelvic nerves and activation of pain-evoked reflexes.

Some risk factors are not modifiable (age, male sex, and type of surgery).

In general, epidural and spinal anesthesia have been associated with higher rates of urinary retention compared with monitored anesthesia care. Opioids or excess intravenous fluid has also been shown to significantly increase the risk of urinary retention. Usually, most problems with urinary retention are self-limited, and will resolve without major intervention. An adequate control of pain is a key point in prevention and treatment. Patients with mild retention are often counseled to sit in a bath of very warm water, filled above the waist. When this is unsuccessful, patients may require bladder catheterization. This may involve intermittent straight catheterization or a temporary indwelling catheter, which can typically be removed after a few days without further testing. Α1 antagonists such as tamsulosin can be helpful, and attempts to minimize opioid intake is also worthwhile [191].

5. Life-threatening complications: Although extremely uncommon, life-threatening complications have been reported after every treatment for hemorrhoids. Surgeons providing hemorrhoid treatment should be aware of the potential serious complications and alert to their presenting features ***(Level of evidence: 1; Grade of recommendation: C)***

Severe septic complications have been reported after all types of treatments of hemorrhoids, and their real incidence is probably underestimated.

Complications such as rectal perforation, pelvic sepsis, abdominal peritonitis, pneumo-retroperitoneum or mediastinum, pulmonary septic embolism, liver abscess, and Fournier's gangrene, with several deaths, have been reported [74, 107, 192, 193].

Several infectious complications have also been reported following office procedures (such as RBL, sclerotherapy, and cryotherapy) including pelvic sepsis, Fournier’s gangrene, liver abscesses, tetanus, and bacterial endocarditis. Deaths due to these infectious complications have been reported too [158].

Even if surgery is usually considered mandatory after serious septic complications, and colostomy often performed, successful conservative treatments (medical, percutaneous drainage) have been reported in selected cases.

The majority of patients in whom these complications occurred were healthy before surgery, and no predisposing factors had been identified. However, it is well known that digital, surgical, or instrumental manipulation of the rectum is associated with a possible 0–9.5% of transient bacteraemia [194], often with no clinical effects. *Escherichia coli* and Bacteroides are the predominant organisms that cause infection following hemorrhoidectomy. The efficacy of a routine use of prophylactic antibiotics has yet to be proven, although special consideration should be given in  immunocompromised patients.

6. Long-term complications: Anal stenosis, soiling, and alterations of anal continence or residual skin tags have been reported after all the treatments for hemorrhoids, without any significant difference among the surgical treatments ***(Level of evidence: 1; Grade of recommendation: B)***

Complications after hemorrhoid surgery are not always immediate, and can instead take months or years to fully develop. In general, they can be more severe and more difficult to treat than those occurring in the immediate postoperative period.

Fifty-one trials (4793 participants; 11 treatments) reported on the proportion of patients complaining of difficulty voiding owing to outlet obstruction or anal stenosis/stricture at follow-up [110].

Anal stenosis has been reported after stapled or excisional hemorrhoidectomy in 1–7.5% of cases [191, 195]. In these patients, the normal pliable anoderm is replaced by scar tissue due to excessive removal of the anoderm and distal rectal or to other factors that interfere with the normal healing process. Concomitant injury of the underlying anal sphincter muscle may also occur and contribute to the functional and anatomical alteration. A functional stenosis, due to muscle hypertonicity, should be considered when planning treatments.

Patients often report straining at defecation, smaller caliber stools, and pain at defecation. Anal stenosis may also lead to fecal impaction and overflow incontinence.

Anal stenosis may be classified, according to the severity of the stricture, as mild, moderate, or severe, but its management is usually determined by the severity of symptoms rather than the degree of stenosis. Mild strictures can often be treated with dietary modifications, stool softeners, or fiber supplements. Digital dilatation or the use of anal dilators can be part of the treatment plan if medical management is not sufficient. Patients with moderate or severe strictures who have failed conservative management usually require surgical intervention. To determine the proper surgical procedure, the degree of involvement of the anoderm and the sphincter muscle complex must be determined. In case of fibrotic anal sphincter, sphincterotomy (unilateral or bilateral) may be considered. On the contrary, patients with stenosis of the anoderm are usually treated with flap (multiple types) or anoplasty, aiming to replace local fibrosis with healthy, elastic tissue [182]. Flap procedures and sphincteroplasty can be associated, in selected patients, while simple release of a stricture may provide temporary relief of symptoms, but generally should be avoided because of the high rate of recurrence.

Fifty-three trials (3856 participants; 9 treatments) reported on the proportion of patients experiencing soiling or difficulty with hygiene or incontinence at follow-up after different types of treatment for HD [110].

Incontinence to feces and/or flatus was reported, with an overall incidence rate of 0.1–17.8%. However, whether this complication was transient or permanent was often not clearly specified [147].

Incontinence after hemorrhoidectomy is usually associated with partial- or full-thickness internal (and occasionally external) anal sphincter injury, but it can occur also with intact sphincters, as the hemorrhoidal cushions are known to provide 15% of the patient’s resting anal tone, and their removal can be functionally disadvantageous. Excision of hemorrhoids with secondary healing may also cause decreased sensitivity and reduced capacity for rectoanal discrimination [196].

Fecal incontinence can also occur after a procedure for prolapse and  HD (reported in up to 5% of patients), and it has been related to a low-placed staple line, to an injury to the internal sphincter due to the large diameter of the circular stapler, or to an alteration of anorectal sensitivity or compliance. In a prospective, randomized trial of 134 patients, de novo fecal incontinence at 1 year was reported in 2.5% of patients who had SH compared with 7.5% of patients who had a Milligan–Morgan hemorrhoidectomy [197].
